# Polycystic Ovary Syndrome: Familiar to Millions?

**DOI:** 10.3390/jcm10010001

**Published:** 2020-12-23

**Authors:** Johannes Ott

**Affiliations:** Clinical Division of Gynecologic Endocrinology and Reproductive Medicine, Medical University of Vienna, 1090 Vienna, Austria; johannes.ott@meduniwien.ac.at

Often, articles about polycystic ovary syndrome (PCOS) start with information about the condition’s high prevalence, the basic characteristics that define this endocrine disorder, and the manifold somatic and/or psychological consequences. Despite the fact that we are all well aware of these circumstances, it still seems important to put an emphasis on the impact of PCOS—not because these facts provide a sound rationale for articles to be published, but because we have to realize that we still cannot provide a good medical solution for a large number of affected women, even though more than 17,500 articles are listed in PubMed^®^ when searching for “polycystic ovary syndrome”. However—and this is a crucial clinical issue—women with PCOS often are unsatisfied with the available pharmaceutical treatment [1]; as widely admitted, these available options either are often contraindicated or associated with unpleasant side effects. For example, contraindications for oral contraceptive pills are common in this patient population, especially because the high prevalence of overweight and obesity [2]. The mentioned side effects often burden women with hypoglycemic agents [3,4] or oral contraception, especially when anti-androgenic progestins are used. 

Empirically, there are two major types of incorrect information that PCOS patients report that they have been provided with. Of course, this might be due to physicians misinforming women or to women’s misunderstanding. The information provided often deals with (i) not being able to conceive at all in the future, which induces panic in the patient, and (ii) complete cure from PCOS. 

Without doubt, the first misinformation is unfortunate and must be avoided. However, I want to focus on the second, since it raises the women’s expectations, which then cannot be fulfilled in the future. PCOS has been called an evolutionary trait which is believed to have persisted for more than 50,000 years. A long time ago, affected women might have benefitted from the condition: early puberty and masculinization might have been a selective force for women and their children in the setting of struggle and recurrent cruelty, and increased bone mineral density and increased muscle mass might have enhanced physical strength. Insulin resistance could have provided glucose availability to protect from starvation, disease, and trauma [5]. Moreover, reproductive longevity might also have contributed to the persistence of PCOS [6]. This sounds highly theoretical but has practical implications: if PCOS is part of the affected women’s “basic configuration”, then only its phenotypic load can be actively modified. In other words, physicians can only help the patients by lessening the symptoms, but cannot make PCOS “disappear”. This needs to be communicated to the affected women in order to form a realistic idea about the whole situation and therefore avoid unnecessary frustration.

Nonetheless, the women we treat for PCOS are in need of diverse, efficient, and well-tolerable treatment options. It should be noted that treatment satisfaction has been mentioned to be an important outcome parameter in studies on PCOS [7]. Thus, future research should not only be about ovarian stimulation, ovulation induction, and artificial reproduction, but also about alleviating hyperandrogenemia-derived symptoms and the (long-term) consequences of hyperinsulinemia and chronic low-grade inflammation, not to forget the psychological burden. Notably, nutritional supplements and herbal medicine have been tested in recent years. So far, only some literature exists and the best evidence is available for inositols and omega three fish oil supplements [8]. Treatment of the metabolic component of PCOS, especially in obese women, will be of great importance. Adiponectin, quercetin, vitamin D, and anti-obesity drugs are among the most promising candidates [9]. The gut microbiota might be a therapeutic target in the future [10]. Moreover, to what extent hypoglycemic agents other than metformin could be of use is an older topic but is still of interest. 

Since only a few ideas found special mention here, one could conclude that PCOS is familiar to millions, but we have to become even more familiar with it. Let us keep up the good work for our patients. Luckily for us researchers, PCOS is really fascinating.

## References

[1] Sills E.S., Perloe M., Tucker M.J., Kaplan C.R., Genton M.G., Schattman G.L. (2001). Diagnostic and treatment characteristics of polycystic ovary syndrome: Descriptive measurements of patient perception and awareness from 657 confidential self-reports. BMC Womens Health.

[2] Diamanti-Kandarakis E., Baillargeon J.P., Iuorno M.J., Jakubowicz D.J., Nestler J.E. (2003). A modern medical quandary: Polycystic ovary syndrome, insulin resistance, and oral contraceptive pills. J. Clin. Endocrinol. Metab..

[3] Legro R.S., Barnhart H.X., Schlaff W.D., Carr B.R., Diamond M.P., Carson S.A., Steinkampf M.P., Coutifaris C., McGovern P.G., Cataldo N.A. (2007). Clomiphene, metformin, or both for infertility in the polycystic ovary syndrome. N. Engl. J. Med..

[4] Tang T., Lord J.M., Norman R.J., Yasmin E., Balen A.H. (2012). Insulin-sensitising drugs (metformin, rosiglitazone, pioglitazone, D-chiro-inositol) for women with polycystic ovary syndrome, oligo amenorrhoea and subfertility. Cochrane Database Syst. Rev..

[5] Ünlütürk U., Sezgin E., Yildiz B.O. (2016). Evolutionary determinants of polycystic ovary syndrome: Part 1. Fertil Steril.

[6] Saxena R., Bjonnes A.C., Georgopoulos N.A., Koika V., Panidis D., Welt C.K. (2015). Gene variants associated with age at menopause are also associated with polycystic ovary syndrome, gonadotrophins and ovarian volume. Hum. Reprod..

[7] Al Wattar B.H., Teede H., Garad R., Franks S., Balen A., Bhide P., Piltonen T., Romualdi D., Laven J., Thondan M. (2020). Harmonising research outcomes for polycystic ovary syndrome: An international multi-stakeholder core outcome set. Hum. Reprod..

[8] Arentz S., Smith C.A., Abbott J., Bensoussan A. (2017). Nutritional supplements and herbal medicines for women with polycystic ovary syndrome; a systematic review and meta-analysis. BMC Complement. Altern. Med..

[9] Saleem F., Rizvi S.W. (2017). New Therapeutic Approaches in Obesity and Metabolic Syndrome Associated with Polycystic Ovary Syndrome. Cureus.

[10] Yurtdaş G., Akdevelioğlu Y. (2019). A New Approach to Polycystic Ovary Syndrome: The Gut Microbiota. J. Am. Coll. Nutr..

